# Blood culture utilization and impact of stewardship practices during a national blood culture bottle shortage at a cancer center

**DOI:** 10.1128/jcm.01160-25

**Published:** 2025-12-08

**Authors:** Rosemary C. She, Jeffrey M. Bender, Vinod Pullarkat, Sanjeet S. Dadwal

**Affiliations:** 1 Department of Pathology, City of Hope Comprehensive Cancer Center 20220 , Duarte, California, USA; 2 Department of Pediatrics, City of Hope Comprehensive Cancer Center 20220 , Duarte, California, USA; 3 Department of Hematology and Hematopoietic Cell Transplantation, City of Hope Comprehensive Cancer Center 20220 , Duarte, California, USA; 4 Department of Medicine, City of Hope Comprehensive Cancer Center 20220 , Duarte, California, USA; Children's Hospital Los Angeles, Los Angeles, California, USA

**Keywords:** persistent fever, test utilization, mortality, sepsis, transplant, diagnostic stewardship

## Abstract

Formulating safe and effective diagnostic stewardship guidance for blood cultures in immunocompromised patients is challenging due to limited data and high risk of infectious complications. A 2024 global shortage in blood culture bottles (BD Diagnostics) necessitated the implementation of blood culture stewardship, including at our tertiary care cancer center. This was a retrospective pre-post intervention study of the effects of diagnostic stewardship at a stand-alone cancer center during a 5-month period of the 2024 blood culture bottle shortage compared to the same 5-month unaffected period in 2023. Interventions included discontinuation of an ordering set for persistent fever, modification of an ordering set for new fever to recommend two sets of blood cultures for initial workup, and issuing revised blood culture ordering guidance to providers, phlebotomists, and nurses with repeated educational efforts. Stewardship interventions led to decreased blood culture utilization by 36.3% (from 14,021 to 8,932), while the number of patients tested remained similar. Greatest reductions in utilization occurred in inpatient encounters and with follow-up blood cultures. There was a trend toward increasing blood culture positivity following intervention, excluding potential contaminants (7.1% vs 7.6%, *P* = 0.08). The frequency of sepsis-coded encounters and 30-day mortality rate were similar between the two time periods. No adverse outcomes related to blood culture stewardship were reported. A significant reduction in blood culture utilization is achievable in a high-complexity cancer patient population without negative clinical impact through limiting the number of initial blood cultures and eliminating pre-emptive ordering of blood culture orders for persistent fever.

**IMPORTANCE:**

Cancer and transplant patient populations are frequently excluded from experimental studies on decreasing blood culture utilization, but a recent global supply shortage in blood culture bottles provided a valuable opportunity to evaluate the effects of stewardship interventions on a large scale. This study, centered on a high-complexity cancer patient population, found that a significant 36.3% reduction in blood culture utilization was achievable, safe, and effective through multidisciplinary diagnostic stewardship efforts.

## INTRODUCTION

Blood culture (BC) ordering practices have come under closer scrutiny as diagnostic and antimicrobial stewardship, and BC bottle supply shortage events have gained attention. Strategies to optimize blood culture utilization in immunocompromised patients are not well studied, and in fact, such patients are often omitted from studies on BC diagnostic stewardship (1, 2). Yet, infectious complications, particularly in hematopoietic stem cell transplant recipients, are a prioritized clinical concern in immunocompromised groups, and BCs are considered a frontline test for workup in febrile neutropenia (3). BCs provide the highest diagnostic yield among microbiological test options (4), and immunocompromised status is frequently included as a criterion in decision support systems to draw BCs considering the increased mortality rates associated with febrile neutropenia (5).

Studies on the clinical impact of implementing diagnostic stewardship for BC ordering in immunocompromised patients are few but suggest that careful optimization can decrease unnecessary BCs in patients with febrile neutropenia without adverse effects. Interventions have included discontinuation of pre-emptive daily BC draws following initial draw or decreasing the frequency of follow-up BCs (6, 7). Best practices for BC in immunocompromised patients, including the number of optimal BCs and use of central venous catheter (CVC) or peripheral blood draws, are not necessarily well defined beyond drawing at least two BC sets on initial workup, and often much is left to clinical discretion (3, 8, 9).

Our objective was to evaluate BC stewardship effectiveness and impact, including potential adverse effects, in a severely immunocompromised cancer population. Given a 2024 global BC bottle shortage (BD Diagnostics, Sparks, MD), many institutions such as ours were required to implement stewardship policies to manage BC bottle inventory. This event provided us with a unique opportunity to better understand and describe the effects of optimizing BC utilization in an exclusively immunocompromised cancer patient population. We hypothesized that thoughtful application of BC stewardship measures would not impact clinical outcomes.

## MATERIALS AND METHODS

This was a retrospective quasi-experimental pre-post intervention study to assess BC utilization and yield at City of Hope (Duarte, CA), a 234-bed comprehensive cancer center with cancer care services that include hematopoietic cell transplant (HCT), chimeric antigen receptor T-cell (CAR-T) therapy, and clinical trials research for predominantly adult and a smaller proportion of pediatric patients. We compared a 5-month period (1 August–31 December 2024) of bottle shortage after implementation of stewardship interventions in July 2024 to the same 5-month period the year before (1 August–31 December 2023). Patients were included if they had a BC collected during the pre-shortage or stewardship implementation period, and the BC was tested in the City of Hope Duarte Clinical Laboratory. BCs that were canceled and not completed were not included in the study.

Pre-BC bottle shortage, typical practice and institutional guidelines included utilization of an electronic order set for new and sudden onset fever (≥38.3°C oral or 38.0°C axillary), which stipulated to draw BCs from all CVC lumens and peripherally; and an order set for persistent fever (>1 fever/24 h) which included drawing BCs from all CVC lumens and peripherally every 24 h for three consecutive days. These order sets were available to all practitioners and not limited to specific clinical conditions. Blood cultures were also available as stand-alone orders. The standard of care practice was that all positive BCs are reviewed by an ID antimicrobial stewardship pharmacist and discussed with an ID physician. Select pathogens (*Staphylococcus aureus*, *Enterococcus* spp., *Pseudomonas aeruginosa*, multidrug-resistant organisms per institutional definitions, *Candida* spp., and non-tuberculous mycobacteria) required a formal ID consultation.

At the onset of the BC bottle shortage, the estimated timeline of shortage provided by the manufacturer and our review of utilization data from previous years informed us that a reduction in BC utilization by 30% was necessary to ensure consistent supply through the shortage period. After meeting with institutional stakeholders from infectious diseases, hematology-oncology, internal medicine, and pediatrics, a set of interventions was implemented in July 2024 in response to the BC bottle shortage: (i) the order set for new and sudden onset fever was revised to stipulate drawing only two BC sets (one BC set being defined as one aerobic and one anaerobic BC bottle for adult patients, or one pediatric bottle for patients <36.3 kg); (ii) the order set for persistent fever was discontinued; and (iii) stewardship recommendations were issued (Table S1) to providers via e-mail bulletins and in-services of phlebotomy and nursing teams. E-mail bulletins were issued approximately monthly thereafter to inform providers of BC bottle inventory and to reiterate our utilization guidelines. In daily operations, phlebotomists disseminated BC shortage guidelines to nursing colleagues on an ongoing basis. Individual cases of potential overutilization were escalated to and addressed by laboratory leadership and infectious diseases physicians. In addition, the microbiology laboratory began to manage BC bottle storage and inventory to track and control utilization, and rationing BC bottles as supplies fluctuated.

Demographic and clinical data were extracted from the electronic medical records on patients with BCs performed during the study period. Data collected included BC specimen-level dates of collection and organisms recovered, patient age, gender, outpatient or inpatient status, history of malignancy, length of stay, receipt of chemotherapy, CAR-T therapy, or HCT during admission, absolute neutrophil count at admission, and mortality 30 days after the last BC. Primary outcome measures of the intervention included reduction achieved in BC order volumes, BC positivity rate, number of BCs per patient tested, inpatient utilization rate per 1,000 patient days, and 30-day all-cause mortality. Sub-analysis was performed on BC episodes, defined as the first BC set drawn every 72 h if previous BCs were negative or at least 14 days after the last positive BC. BC contamination rates were calculated using standard laboratory definitions (10). To compare the complement of organisms recovered in BCs, monomicrobial BC data were collected, counting each BC that recovered a given organism for a given patient in each study period.

Quantitative data are summarized as means with standard deviations when normally distributed and as medians with interquartile ranges when not normally distributed. The chi-square method was used to compare qualitative outcomes between groups, and the chi-square test of homogeneity was used to compare distributions of proportions of two or more categories between populations. For quantitative data comparisons, the Mann-Whitney *U* test was applied for non-parametric distribution and the *t*-test for parametric data. Statistical analyses were performed with two-tailed comparisons, and *P* values of <0.05 were considered significant, using GraphPad Prism, version 10.4.2.

## RESULTS

### Patient and blood culture baseline characteristics

In both periods, the median age of patients who had BCs was 61 years, and approximately half were male (Table 1). Over 98% of patients had a history of malignancy, with hematologic malignancies representing slightly over half of all patients who had BCs. Approximately 40% of patients had received chemotherapy, and 9.5%–13.0% were recipients of HCT or CAR-T during hospitalization. Despite statistically significant differences in clinical characteristics of patients with BCs between 2023 and 2024, the number of sepsis-coded admissions remained unchanged. Patients with BCs in the 2023 group had longer lengths of stay, higher numbers with hematologic malignancies, and higher numbers with HCT/CAR-T during hospitalization; however, during the 2024 shortage period, there were higher numbers of admissions with BCs ordered. Thirty-day mortality (after the most recent BC was taken) was similar between groups: 13.9% in 2023 and 14.1% in 2024 (*P* = 0.83). No adverse events related to BCs were reported to the hospital quality program during the 2024 study period.

**TABLE 1:** Patient and clinical characteristics, pre- vs post-BC shortage groups

	Pre-BC shortage (2023)	During BC shortage (2024)	*P* value
Unique patients, *n*	1,920	1,993	
Male, *n* (%)	1,032 (53.8)	1,033 (51.8)	0.23
Age (yr), median (IQR)	61.7 (49.6–70.1)	61.9 (49.6–70.8)	0.36
Patients <18 yr, *n* (%)	28 (1.5)	19 (1.0)	0.15
History of malignancy, category, *n* (%)			0.04
Hematologic malignancy	987 (51.4)	968 (48.6)	0.08
Solid organ malignancy	824 (42.9)	922 (46.3)	0.04
Hematologic and solid organ malignancy	83 (4.3)	66 (3.3)	0.10
No malignancy	26 (1.4)	37 (1.9)	0.21
30-day mortality after last BC, *n* (%)	267 (13.9)	282 (14.1)	0.83
Outpatient visits with BCs ordered	890	954	
Admissions with BCs ordered	2,073	2,225	
Unique inpatients, *n*	1,598	1,647	
Length of stay (days), median (IQR)	7 (4–18)	6 (3–14)	<0.01
CAR-T during admission, *n* (%)	53 (2.6)	47 (2.1)	0.33
HCT during admission, *n* (%)	216 (10.4)	164 (7.4)	<0.01
Chemotherapy during admission, *n* (%)	903 (43.6)	840 (37.8)	<0.01
Absolute neutrophil count at admission <500/µL, *n* (% of admits)	328 (15.8)	248 (11.1)	<0.01
Sepsis-coded encounter within 7 days of BC, *n* (%)	375 (18.1)	381 (17.1)	0.41

While the number of unique patients who had BCs performed was slightly higher in the 2024 period (1,920 in 2023 vs 1,993 in 2024), BC utilization decreased by 36.3%, from 14,021 to 8,932 BCs, during the shortage period. Reduced utilization was also reflected by significantly fewer BCs performed per patient during the shortage period (mean 4.5 in 2024 vs 7.3 in 2023, *P* < 0.01). A decrease in utilization was largely observed in hospitalized patients, whereas outpatient utilization was unchanged between the two time periods (Table 2).

**TABLE 2:** Blood culture utilization and yield, pre- and post-BC bottle shortage

	Pre-BC shortage (2023)	During BC shortage (2024)	*P* value^*f*^
Blood cultures performed, *n*	14,021	8,932	
Type of BC draw, *n* (%)			<0.01
BC peripheral draw	6,075 (43.3)	3,590 (40.2)	<0.01
BC line draw	7,418 (52.9)	4,929 (55.2)	<0.01
BC draw type not specified	528 (3.8)	413 (4.6)	<0.01
Mean number of BCs per patient during the study period	7.3	4.5	<0.01
Positive BC, *n* (%)	1,073 (7.7)	752 (8.4)	0.04
Contaminated BC, *n* (%)	73 (0.52)	59 (0.66)	0.17^*a*^
All others, *n* (%)	1,000 (7.1)	693 (7.6)	0.08^*a*^
Outpatient BCs, *n* (% positive)	1,649 (5.5)	1,637 (6.3)	0.35^*a*^
Positive BC excluding contaminants, *n* (%)	81 (4.9)	97 (5.9)	0.20^*a*^
Inpatient BCs, *n* (% positive)	12,372 (7.9)	7,295 (8.9)	0.02^*a*^
Positive BC excluding contaminants, *n* (%)	919 (7.4)	598 (8.2)	0.051^*a*^
No. of inpatient BC per 1,000 patient-days	359.7	223.0	<0.05
Unique patients with positive BC, *n*	330	296	
Unique patients with positive BC excluding CTM, *n*	283	261	
Repeat BC positive within 14 days	595	360	
BC episodes, *n*^*b*^	3,281	3,562	
BC positive on initial day, *n* (%)^*c*^	312 (9.5)	300 (8.4)	0.12
BC drawn on day 2	783	260	
BC positive for new organism, day 2, *n* (%)	15 (1.9)^*d*^	4 (1.5)	0.69^*a*^
BC drawn on day 3	757	405	
BC positive for new organism, day 3, n (%)	18 (2.4)^*e*^	11 (2.7)^*e*^	0.72^*a*^
No. of BC sets drawn on initial day of episode, *n* (%)			<0.01
1 set	367 (11.2)	581 (16.3)	<0.01
2 sets	1,746 (53.2)	2,586 (72.6)	<0.01
3 sets	1,035 (31.5)	350 (9.8)	<0.01
4 sets	102 (3.1)	42 (1.2)	<0.01
5 sets	24 (0.7)	3 (0.1)	<0.01
6 sets	8 (0.2)	0	<0.01
BC yield day 1 of episode by no. of sets drawn, *n* (%)^*e*^			
1 set	20 (5.4)	20 (3.4)	
2 sets	157 (9.1)	257 (9.9)	
3 sets	147 (14.2)	64 (18.3)	
4 sets	18 (17.6)	7 (16.7)	

^*a*^ Represents *P* value for comparing the percentage positive between 2023 and 2024.

^*b*^ Episode defined as BC sets drawn over a 72 h period and no less than 72 h after the previous negative BC or 14 days after the last positive BC.

^*c*^ Excludes 44 contaminated BCs in the 2023 group and 49 contaminated BCs in the 2024 group.

^*d*^ Excludes five contaminated BCs. No BCs in this category for the 2024 group were contaminated.

^*e*^ Excludes seven contaminated BCs in the 2023 group and two contaminated BCs in the 2024 group.

^*f*^ *P *< 0.01 by chi-square analysis for the no. of BC sets vs BC yield, 2023 and 2024 data combined.

### BC organism recovery and changes in ordering practice

The overall BC positive rate was significantly higher during the shortage (8.4% vs 7.7% in 2023, *P* = 0.04), but this difference lost statistical significance once potential BC contaminants are excluded (7.6% vs 7.1%, *P* = 0.08). When analyzed separately, the BC positive rate among inpatients but not outpatient BC draws was significantly higher (Table 2). The number of unique patients with positive BCs was lower during the shortage period (296 in 2024 vs 330 in 2023), though the difference is less when including only patients with potential pathogens rather than contaminants isolated (283 in 2023 and 261 in 2024). The number of positive BCs after excluding repeat positives was lower during the 2024 than 2023 period (

395 vs 486, respectively) (Table 2). BC contamination rates did not differ significantly between periods even though a slightly higher proportion of BCs were obtained through CVC during the 2024 shortage. Monomicrobial BCs were analyzed and demonstrated a higher number of isolates across most microorganism groups pre-shortage vs shortage period, without any consistent trends noted (Fig. S1). There were also more polymicrobial BCs, defined as two or more organisms recovered within a 72 h period, in 2023 (*n* = 64) than in 2024 (*n* = 45), though the proportion to all positive BC episodes was similar (13.3% vs 11.4% respectively, *P* = 0.40).

By BC episodes, there were 3,561 during the 2024 shortage compared to 3,281 pre-shortage period. Prior to the bottle shortage, three or more BC sets were drawn on 35.5% of the initial fever workup. During the shortage period, this declined to 12.0% and practice shifted toward drawing two sets for the initial workup (78.7% compared to 53.3% in 2023) (Table 2). Across both periods, organism recovery depended on the number of BC sets drawn during initial 24 h of fever workup and was highest with three to four BC sets (14.1%–18.3%) compared to two sets (9.1%–9.9%) or one set (3.4%–5.4%) (*P* < 0.01).

Within BC episodes, 59 total cases across both time frames had additional BC recoveries on Day 2 and/or Day 3 BC draws compared to Day 1 BC alone. After excluding likely contaminants (11 cases), recovery of additional microorganisms was observed in 1.5%–1.9% of Day 2 and 2.4%–2.7% of Day 3 BCs, with no statistically significant differences between years (Table 2, Tables S2 and S3). Of these 48 remaining cases, 30 (62.5%) represented polymicrobial infections in which a different pathogen was recovered on Day 2 or 3 than the initial Day 1 BCs. Follow-up cultures on Day 2 or 3 after initial BCs on Day 1 of an episode were performed in significantly fewer cases during the 2024 shortage period and more frequently as follow-up of positive Day 1 BCs (Table S4). No change in antimicrobial therapy was made in response to additional Day 2–3 BC organisms in 40.5% of 2023 and 31.3% of 2024 cases (Table 3). Most of these cases that had Day 2–3 BCs were drawn for surveillance purposes rather than change in clinical status. For cases in which antimicrobial coverage was broadened or switched, over half had Day 2–3 BCs drawn due to persistent fever, new fever, or worsening clinical status. The remainder were drawn for surveillance purposes, recovering potential pathogens in 19% and contaminants in a smaller proportion (9.5% in 2023 and 12.5% in 2024) that were treated with antimicrobial agents out of caution.

**TABLE 3:** Antimicrobial therapy in response to additional organism detections in BCs on day 2 or 3 of BC episode^*a*^

	Pre-BC shortage (2023) (*n* = 42)	During BC shortage (2024) (*n* = 16)
No change in antibiotic regimen	17 (40.5%)	5 (31.3%)
D2/D3 BCs performed for surveillance, clinically stable	7 (16.7%)	4 (25.0%)
D2/D3 BCs performed for surveillance, contaminant recovered	7 (16.7%)	1 (8.3%)
Patient receiving end of life care, expired before final BC results	3 (7.1%)	0
Antimicrobial coverage broadened or adjusted	25 (59.5%)	11 (68.8%)
D2/D3 BCs drawn for worsening clinical status or new fever	10 (23.8%)	4 (25.0%)
D2/D3 BCs drawn for persistent fever	3 (7.1%)	2 (12.5%)
D2/D3 BCs performed for surveillance, clinically stable	8 (19.0%)	3 (18.8%)
D2/D3 BCs performed for surveillance, contaminant recovered and treated	4 (9.5%)	2 (12.5%)

^*a*^ BC, blood culture; D2, day 2; D3, day 3.

## DISCUSSION

The data presented here demonstrated that during a critical, months-long BC bottle shortage, diagnostic stewardship interventions in BC ordering were both effective and safe in a highly immunocompromised patient population. Our cancer center provides care for a patient population that is among those with the highest case-mix index, indicating high-complexity and resource-intensive inpatient cases (11). Despite the higher level of care required, our site was able to achieve a decrease in BC utilization by one-third of the previous usage, while mortality rates among patients requiring BCs remained remarkably stable across pre- and post-intervention periods. The decreased utilization of BC bottles was achieved while accommodating a slightly higher number of patients requiring BCs and hospitalizations compared to the pre-intervention period. Unsurprisingly, the impact in utilization was much more pronounced in hospitalized patients, who may have multiple BCs ordered during hospitalization, than in outpatient encounters. While we cannot fully account for the variables involved in the BC results and patient outcomes, these real-world data during a BC supply shortage are encouraging with regard to application of diagnostic stewardship in an immunocompromised, cancer patient population.

The small increase in BC positive rate during the shortage was disproportionately associated with a major decrease in numbers of BCs tested, suggesting more efficient utilization of BCs. Target BC positive rates, while not narrowly defined, have been recommended to fall within the 5%–15% range, with a national audit demonstrating a mean of ~8% by institution (12, 13). The BC positive rate in both periods at our institution fell around 8% with little difference between years, suggesting appropriate BC utilization. Enumerating inpatient BCs per 1,000 patient-days is another metric of BC utilization (12), and attempts have been made to establish thresholds for this metric for intensive care unit (ICU), medical ward, and surgical unit populations. Suggested minimum thresholds based on nationally surveyed hospitals vary from 30 to 120 BCs/1,000 patient-days, depending on unit type (14, 15), while individual diagnostic stewardship studies have reported ranges up to 228 BCs/1,000 patient-days (16). Importantly, site-specific BC needs are likely to hamper meaningful standardization of this metric (14). There are few data to aid in proposing an optimal range for cancer centers, but we were able to reduce the figure from 360 BCs/1,000 patient-days to 223 BCs/1,000 patient-days, close to the upper end reported in medical ICUs. Additional data are needed to better understand if optimal BC utilization differs in cancer and other immunocompromised patient populations.

Institutions using BD blood culture systems were variably impacted by the bottle shortage in 2024, with some having to implement extreme protocols on BC ordering beyond the standard of care during the crisis (17, 18). Other studies have reported that the use of information technology (IT) interventions such as hard stops and clinical decision support tools for test ordering, especially when the number of BC orders exceeded a certain number per day, was effective in supporting stewardship interventions, reducing BC utilization by 30%–40% (17, 19, 20). Our findings were in line with these reduction rates and made possible by positive collaborations between laboratory, infectious diseases providers, IT specialists, and hematology-oncology providers to construct a consensus strategy and culture change to manage BC utilization during the shortage. The rapid IT response to discontinue the highly utilized ordering set for persistent fever had an immediate impact. Through close daily monitoring of BC bottle inventory and early data review of organism yield for three vs two BC sets at our site, we decided against implementing IT hard stops for BC ordering and to allow provider discretion for ordering BCs outside of the temporary stewardship guidance. Individual cases of overutilization were addressed by laboratory personnel or infectious disease consultation. The data summarized here reflect the widely accepted finding that three to four BC sets provide higher recovery of bloodstream pathogens than two BC sets (21, 22), although we acknowledge that in our retrospective analysis, the ordering of three BCs rather than two may itself be associated with the heightened suspicion and higher pre-test probability of bloodstream infection.

In support of NCCN guidelines and studies on the value of repeat BCs in cancer patients, BCs drawn routinely after the first 24 h of presentation provided little benefit (3, 7, 23–25). Consistent with these studies, our data showed only 1%–3% incremental increases in organism recovery over initial BC draws when repeat BCs are drawn over the following 2 days. Furthermore, more than half of these infrequent additional organism recoveries occurred when the initial BCs had isolated a different organism. Another concern that may be overlooked in such overutilization of BCs is iatrogenic anemia in a patient population that is frequently pancytopenic and receiving blood transfusion to support hemodynamic status. Each blood culture requires 8–10 mL of blood to be drawn into each bottle, with one standard blood culture consisting of two bottles (one aerobic and one anaerobic). Three blood cultures, therefore, require 48–60 mL of blood to be drawn from the patient. Our intervention reduced the occurrence of episodes in which more than five blood culture sets were obtained within the first 24 h to nearly zero in the stewardship period.

There are limitations to this study. Primarily, this is a retrospective design which, while providing large numbers of BC data at a cancer center, could not control for variation in the case mix, which is expected to occur over time (11). The 2023 study period saw increased numbers of HCT, neutropenia, and chemotherapy, which should be considered when interpreting the results. However, sepsis-coded encounters did not differ significantly between time periods, which suggests the time periods were similar in terms of proportion of patients at risk of severe bloodstream infection. Data on antimicrobial usage or days on antimicrobial therapy were not collected; therefore, it is unknown if such variables affected the study findings. As the time frame of the BC bottle shortage was limited, the sample size could not be powered sufficiently to find statistical significance in outcomes with very small observed differences, such as 30-day mortality. The study intended to capture the implementation of diagnostic stewardship for BC across an entire cancer center patient population and did not specifically focus on certain patient groups such as those with neutropenic fever, areas in which focused diagnostic stewardship studies are needed. We also did not focus on impact to antimicrobial stewardship, infection control practices, or timing of CVC removal in cases of suspected line infection as a result of our stewardship intervention, though these areas may be worthwhile for future investigations. It should be noted that there was likely overutilization of BCs at our site prior to the intervention, which facilitated reduction of BC usage without adverse outcomes.

Post-BC shortage, we have kept many of the interventions implemented in place, including eliminating the daily blood culture order sets and updating institutional guidelines to limit blood cultures to a maximum of three sets (Table 4). We did observe a small rebound in BC order volumes, but BC utilization remained steadily 23% lower over a recent 5-month period after the BC bottle shortage than the same period before the shortage (data now shown).

**TABLE 4:** BC ordering recommendations pre-intervention, during the BC bottle shortage, and after the shortage had resolved^*a*^

Clinical scenario	Pre-intervention BC practices	Recommendations during BC bottle shortage	Recommendations post-BC bottle shortage
New-onset fevers	Draw BCs from all ports of the patient’s VAD and a peripheral vein (also an order set).	Draw no more than two BCs (order set updated).	Draw two to three BCs (order set updated).
Persistent fever	Draw BCs from all ports of the patient’s VAD and a peripheral vein (if no VAD, BCs from two peripheral sites) every 24 h for 3 days (also an order set).	Draw two BCs if initial BCs show no growth after 48 h (order set discontinued).	Draw two BCs if initial BCs show no growth after 24 h.
Follow-up of positive initial BC	Collect daily BCs until BCs have become negative.	Collect BCs no earlier than 72 h later (96 h for fungemia).	Collect BCs no earlier than 48 h later.

^*a*^ BC, blood culture; VAD, vascular access device.

In summary, this study describes the diagnostic stewardship intervention for BCs to be safe and effective in significantly altering BC ordering patterns and reducing BC utilization in a highly immunocompromised patient population. While at least two BCs are recommended for initial workup of bloodstream infection, three BCs can provide optimal yield, especially when bloodstream infection is highly suspected. Repeating blood cultures within 48 h of initial workup has very low yield and should not be routinely performed even in patients with malignancy unless clinically indicated.

## SUPPLEMENTAL MATERIAL

Supplemental Material jcm.01160-25-s0001.docx Tables S1 to S4; Figure S1.

ASM does not own the copyrights to Supplemental Material that may be linked to, or accessed through, an article. The authors have granted ASM a non-exclusive, world-wide license to publish the Supplemental Material files. Please contact the corresponding author directly for reuse.
