# Supplementary material for: Blood culture utilization and impact of stewardship practices during a national blood culture bottle shortage at a cancer center
Source: J Clin Microbiol. 2025 Dec 8;64(1):e01160-25. doi: 10.1128/jcm.01160-25 (PMC12802142; doi:10.1128/jcm.01160-25)
Supplement: Supplemental Material — Tables S1 to S4; Figure S1. [file jcm.01160-25-s0001.docx]

**Supplemental data**

**Table S1**. Blood culture utilization guidelines used to educate providers via lab alert emails and phlebotomy and nursing in-servicing education at our campus.

| - **Please limit to 2 blood cultures**per patient for initial diagnostic work-up or for persistent fever. We continue to receive 3 or more blood cultures being collected at a time for some patients and this practice is unsustainable in the current shortage. - "Blood Culture for Persistent Fevers" Order Set has been discontinued as of 7/17/2024. **Please do not activate this Order Set**if it was placed as a standing order prior to 7/17/2024. - **All blood cultures should be ordered only if there is evidence or high suspicion of blood stream infection/sepsis, and not preemptively/for surveillance.** - For optimal organism recovery and results, follow collection site disinfection protocol and **draw 8-10 mL of blood per bottle.** - For **initial**diagnostic work-up of bloodstream infection/sepsis, please **limit the number of blood cultures to 2**(one blood culture consists of 1 aerobic bottle and 1 anaerobic bottle). Draw each blood culture from a different site:   - In patients with central line, draw from **2 different lumens.**   - In patients without central line, perform **2 peripheral draws from separate sites**. - In the case of **persistent fever in the HCT recipient**, **only 2 additional sets of blood cultures** should be drawn if the initial blood culture sets show no growth **after 48 hours.** - Beyond this initial evaluation, additional repeat blood cultures should only be ordered if there is a change in clinical status. - After an initial positive blood culture, any follow-up blood cultures should be drawn **no earlier than 72 hours (or 96 hours for fungemia),** and preferably under guidance of Infectious Disease. |
| --- |

**Table S2.** Pre-BC shortage (2023) cases demonstrating additional organisms detected on day 2 and day 3 BCs with comparison to day 1 BC findings, within BC work-up episodes.

| *n* | Day 1 | Day 2 | Day 3 |
| --- | --- | --- | --- |
| 25 | No growth | 14 No growth or Not done | 5 CoNS (includes 3 CTM) |
|  |  |  | 1 MCR *Paenibacillus* sp. (CTM) |
|  |  |  | 3 NF-GNR |
|  |  |  | 2 Viridans group streptococcus |
|  |  |  | 1 *Bacillus cereus* group |
|  |  |  | 1 *Enterococcus faecium* |
|  |  |  | 1 Enteric GNR |
|  |  |  | 1 *Histoplasma capsulatum* |
|  |  | 4 CoNS (CTM) | 4 No growth or Not done |
|  |  | 4 Enteric GNR | 4 No growth |
|  |  | 1 *Candida albicans* | *Enterococcus faecium* |
|  |  | 1 *Lactobacillus species* | Not done |
|  |  | 1 *Enterococcus faecalis* | No growth |
| 4 | Enteric GNR | 2 No growth or Not done | 1 *Enterococcus faecium* |
|  |  |  | 1 CoNS |
|  |  | 2 NF-GNR | 2 Not done |
| 3 | NF-GNR | 2 No growth or Not done | 1 *Streptococcus agalactiae* |
|  |  |  | 1 *Enterococcus faecium* |
|  |  | 1 CoNS | No growth |
| 2 | Mixed NF-GNR | 1 NF-GNR #4 | 1 NF-GNR #4 |
|  |  | 1 No growth | *Brevibacterium* species |
| 3 | Mixed GN and GP | 1 *Enterococcus faecium* | Not done |
|  |  | 1 CoNS (CTM) | *Leuconostoc* sp. |
|  |  | 1 No growth | *Trichosporon asahii* |
| 1 | Viridans streptococci | *Candida albicans* | *Candida albicans* |
| 1 | Coryneform rod (CTM) | *Candida glabrata* | Not done |
| 2 | *Enterococcus faecium* | 1 Enteric GNR | Not done |
|  |  | 1 Not done | CoNS |
| 2 | CoNS | 1 *Candida glabrata* | No growth |
|  |  | 1 Not done | *Candida guilliermondii* |

Abbreviations: CoNS, coagulase-negative *Staphylococcus*; CTM, contaminant based on CLSI M47 [10]; GN, Gram-negative; GP, Gram-positive; GNR, Gram-negative rod; NF, Non-Fermenter; MCR, most closely resembles.

**Table S3.** BC bottle shortage period (2024) cases demonstrating additional organisms detected on day 2 and day 3 BCs with comparison to day 1 BC findings, within BC work-up episodes.

| *n* | Day 1 | Day 2 | Day 3 |
| --- | --- | --- | --- |
| 4 | Enteric GNR | 2 Not done | 1 Enteric GNR #2 |
|  |  |  | 1 *Bifidobacterium* species |
|  |  | 1 Enteric GNR #2 | Not done |
|  |  | 1 CoNS | CoNS |
| 1 | Mixed Enteric GNR | No growth | *Candida glabrata* |
| 3 | *Candida* sp. | Not done | 1 *Candida lusitaniae* |
|  |  |  | 1 CoNS (CTM) |
|  |  |  | 1 *Stenotrophomonas maltophilia* |
| 3 | No growth | Not done | 1 *Clostridium perfringens* |
|  |  |  | 1 Enteric GNR |
|  |  |  | 1 *Corynebacterium* sp. (CTM) |
| 2 | NF-GNR | Not done | 1 CoNS |
|  |  |  | 1 NF-GNR #2 |
| 1 | Mixed GN and GP | NF-GNR | Not done |
| 1 | *Enterococcus faecium* | Not done | NF-GNR |
| 1 | CoNS | *Fusobacterium nucleatum* | No growth |

Abbreviations: CoNS, coagulase-negative *Staphylococcus*; CTM, contaminant based on CLSI M47 [10]. GN, Gram-negative; GP, Gram-positive; GNR, Gram-negative rod; NF, Non-Fermenter.

**Table S4.** Number of repeat BCs performed on day 2 or 3 based on results of initial (day 1) BCs.

|  | **2023** | **2024** | **P-value** |
| --- | --- | --- | --- |
| Day 2 BC drawn | 784 | 260 |  |
| When Day 1 BC positive | 199 | 127 | <0.01 |
| When Day 1 BC negative | 585 | 133 |  |
| Day 3 BC drawn | 758 | 405 |  |
| When Day 1 BC positive | 221 | 153 | <0.01 |
| When Day 1 BC negative | 537 | 252 |  |
| When Day 2 BC positive | 10 | 3 | 0.21 |
| When Day 1 BC negative | 525 | 70 |  |

**Supplemental Figures**

**Sup. Figure 1.** Comparison of recovery of monomicrobial blood culture organisms between 2023 (n=417 pre- and 2024 post- (n=349) blood culture shortage intervention. Data shown represent unique counts; contaminants were counted separately and not included in other categories.

Abbreviations: NF-GNR, Non-fermenting Gram-negative rod; CoNS, coagulase-negative S*taphylococcus*; GPC, Gram-positive cocci; GPR, Gram-positive rod.
